# Mechanism and *In Vivo* Evaluation: Photodynamic Antibacterial Chemotherapy of Lysine-Porphyrin Conjugate

**DOI:** 10.3389/fmicb.2016.00242

**Published:** 2016-03-02

**Authors:** Zengping Xu, Yuxiang Gao, Shuai Meng, Baochen Yang, Liyun Pang, Chen Wang, Tianjun Liu

**Affiliations:** ^1^Tianjin Key Laboratory of Biomedical Material, Institute of Biomedical Engineering, Peking Union Medical College – Chinese Academy of Medical SciencesTianjin, China; ^2^Department of Materials Chemistry, Nankai UniversityTianjin, China; ^3^Tianjin Medical University Cancer Institute and HospitalTianjin, China

**Keywords:** lysine-porphyrin conjugate, photodynamic antimicrobial chemotherapy, antibiotic resistance, photosensitizer, mixed bacterial-infected wounds

## Abstract

Lysine-porphyrin conjugate 4i has potent photosensitive antibacterial effect on clinical isolated bacterial strains such as Methicillin-resistant *Staphylococcus aureus* (MRSA), *Escherichia coli*, and *Pseudomonas aeruginosa*. The mechanism of photodynamic antibacterial chemotherapy of 4i (4i-PACT) *in vitro* and the treatment effect *in vivo* was investigated in this paper. Atomic force microscopy (AFM) revealed that 4i-PACT can effectively destroy membrane and wall of bacteria, resulting in leakage of its content. This was confirmed by dual fluorescent staining with acridine orange/ethidium bromide and measuring materials absorption at 260 nm. Agarose gel electrophoresis measurement showed that 4i-PACT can damage genomic DNA. Healing of wound in rat infected by mixed bacteria showed that the efficiency of 4i-PACT is dependent on the dose of light. These results showed that 4i-PACT has promising bactericidal effect both *in vitro* and *in vivo*.

## Introduction

The antibiotics for treating common infections have become less effective in recent years. Nearly 1000 of resistance-related β-lactamases, which inactivate the antibiotics, have been identified, a 10 times increase since 1990 (Davies and Davies, 2010; Laxminarayan et al., 2013). This situation made us at the dawn of post antibiotic era (Ryono, 2014). So, it is urgent to find alternative approaches against antibiotics-resistant bacteria. Generally, there are two solutions to this problem. One is to develop novel antibacterial agents, the other is to develop novel physical sterilization. The latter includes radiation sterilization (Nusbaum and Rose, 1979), high pressure electric field sterilization (Sasagawa et al., 2006), microwave sterilization (Dovigo et al., 2009), magnetic sterilization (Haile et al., 2008), and pulse light sterilization (Krishnamurthy et al., 2004). Chemical agents and physical approaches are combined in Photodynamic antimicrobial chemotherapy (PACT) (Wainwright, 1998), which consists of a non-toxic photosensitizer (PS), illumination light of an appropriate wavelength, and ambient molecular oxygen. However, not all of the PS can be used in PACT treatment of bactrerial infection. Only special designed PS can match the PACT demand (Meng et al., 2015). So from this point of view, PACT falls between novel antibacterial agents and novel physical sterilization approaches, namely, a physicochemical treatment. With the advantage of circumventing the bacterial multidrug resistance in the treatment of infection, PACT has received great attention in recent years.

The PS is the crutial element in PACT. In the past few decades, several types of PSs have been reported, such as porphyrin, heme, phthalocyanine, phenothiazinium dyes, BODIPY, methylene blue, conjugated polyelectrolytes, cationic functionalized fullerene, as well as nanoparticals (Wainwright and Crossley, 2004; Wainwright, 2010; Fu et al., 2013; Sperandio et al., 2013). As an ideal PS candidate for photodynamic therapies (Stojiljkovic et al., 2001), Porphyrin can efficiently kill Gram-positive bacteria. Meanwhile, in combination with agents that permeabilize the highly organized outer membrane of Gram-negative bacteria, only cationic PSs, or noncationic PSs are able to kill Gram-negative species (Mbakidi et al., 2013; Prasanth et al., 2014).

In a previous paper, we have reported the synthesis, characterization, and *in vitro* photodynamic antimicrobial activity of basic amino acid-porphyrin conjugates (Meng et al., 2015), a new class of PSs. Among them, compared with other PSs, 4i exhibits a broader spectrum of photoinactivation *in vitro*, improved biocompatibility and stability (Bertolini et al., 1990; Nitzan et al., 1992; Tomé et al., 2004; Liu et al., 2012; Gomes et al., 2013; Mbakidi et al., 2013; Prasanth et al., 2014). This paper reported the mechanism of bacterial inactivation by 4i-PACT and its effectiveness *in vivo* on experimental mixed bacterial infected rat wounds. The results showed that 4i-PACT had potent bactericidal effect both *in vitro* and *in vivo*.

## Materials and Methods

### Bacterial Culture

In this experiment, three clinical bacterial strains, MRSA, *Escherichia Coli*, and *Pseudomonas aeruginosa* were isolated at the Tianjin Armed Police Hospital. These bacterial strains were cultured in Luria Bertani (LB) medium. A single colony was used to inoculate 10 mL of liquid medium. The bacteria was grown under aerobic condition at 37°C in a shaking incubator (200 rpm) for 18 h. The bacteria was then collected by centrifugation and resuspended in an equal volume of PBS (Zhao et al., 2014).

### Chemicals and Instruments

**FIGURE 1:**
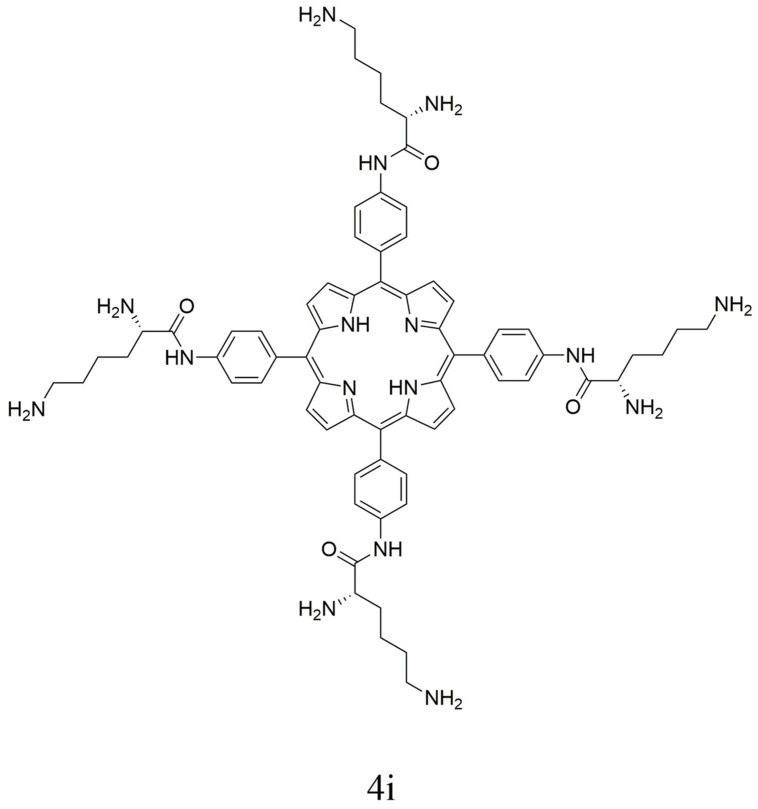
**Structure of 5,10,15,20-trakis(4-((*S*)-2,6-diaminohexanamido)-phenyl) porphyrin (4i)**.

Porphyrin derivative 4i was synthesized and characterized with the procedure reported by Meng et al. (2015). For clarity, synthetic procedure and characterization detail are omitted. The chemical structure of porphyrin derivative 4i is shown in **Figure 1**.

Light from a semiconductor laser (7404, Intense, USA) with a wavelength of 650 nm was delivered on the sample via an optic fiber. The energy density of light spot was measured by a light power meter (LM1; Carl Zeiss).

### MIC and MBC Determinations

Three bacterial strains were treated with the same procedure. As an example of all of the strains, the treatment of MRSA is described in detail. Experiments were performed in 96-well flat bottom plates. Twenty microliter of MRSA suspension and 180 μL of the compounds were added to each well. The final concentration of the bacterial in the mixture was 10^6^ colony-forming units/mL (CFU/mL). 4i with different mole concentrations were prepared, e.g., 0.98, 1.95, 3.91, 7.8, 15.6, 31.25, 62.5, 125, 250, and 500 μM, respectively. Plates were kept in the dark for 30 min at 37°C and then exposed to light illumination for 30 min or kept in the dark to provide dark control samples. After light exposure, the samples were incubated in dark at 37°C and CFU was evaluated after 18 h. Three sets of independent experiments were performed (Rodrigues et al., 2013).

### Dose-Dependent Photoinactivation Effects

Suspensions of bacteria (10^7^ CFU/mL) were incubated at 37°C for 30 min in the dark with 4i (3.91, 7.81, 15.63, 31.25, and 62.50 μM). The suspensions were then loaded into a 96-well plate and illuminated with light with energy density of 6 J/cm^2^ for the photoreaction. After illumination, 100 μL mixture from each well was taken to determine the CFU. The mixtures were serially diluted to 10^1^, 10^2^, 10^3^, 10^4^, and 10^5^ times of the initial concentrations using PBS. 100 μL of each dilution was plated onto LB agar plates and incubated for 18 h at 37°C in dark. The CFUs were counted, and each experiment was performed in triplicate. The surviving fractions of bacteria were expressed as ratios of CFU produced by bacteria treated with PSs and light (Grinholc et al., 2008).

### Atomic Force Microscopy (AFM) Imaging

The bacteria were examined with atomic force microscope (Veeco Multi Mode 8/B0021, Veeco German) equipped with Antimony doped silicon of conical shape (Veeco). The force constant is 3 N/m. Bacterial samples in a concentration of 10^7^ CFU/mL were dropped on to the mica plate surface (about 0.5 cm^2^), naturally dried at ambient temperature, then were scanned in intelligent mode (Wu et al., 2014).

### Fluorescence Imaging

In order to study 4i-PACT effect on the permeability of the bacterial membrane, the samples after 4i-PACT treatment were stained with 4 μL of the acridine orange/ethidium bromide (AO/EB) double fluorescent dyes mixture (100 mg/L of AO and 100 mg/L of EB) for 5 min in dark. Then 5 μL of the stained mixture was dropped on a microscopic glass slide with cover glass and immediately checked by the fluorescent microscope (Nikon Eclipse Ti/B0004, Nikon, Japan) at 200× magnification (Sui et al., 2013).

### Genomic DNA Extraction and Electrophoresis

Bacterial suspensions (1 × 10^7^ CFU/mL) were treated with 10 μM 4i as mentioned in experiment 2.4 above. After exposing to the laser light, the genomic DNA was extracted from bacteria immediately using a Genomic DNA Purification Kit (Solarbio, Beijing). DNA samples were gently mixed with 6x loading buffer (Solarbio, Beijing). DNA was analyzed by electrophoresis with 0.8% agarose gel in 1x TAE buffer at 70 V for 30 min. DNA green was incorporated into the agarose gel. The 15000 bp DNA Marker (TAKARA) was used as a molecular weight marker with DNA fragments between 250 and 15000 bp. The light dose had been optimized *in vitro* in our experiments. Light dose higher than 6 J/cm^2^ did not improve the efficacy, while the light with lower dose results in a lower bactericidal ability. Without PS 4i, light exposure alone on bacteria *in vitro* did not have significant bactericidal effect.

### Exudation Study for Measuring Materials Absorption at 260 nm

DNA and RNA have absorbance at 260 nm, so exudation study was conducted by measuring the materials absorption at 260 nm for bacteria treated by 10 μM 4i-PACT, 10 μM 4i alone, and PBS, respectively. After treatment, 400 μL of the bacterial suspensions (1 × 10^8^) were filtered to remove the bacteria. OD at 260 nm (OD_260_) of the supernatant was recorded for treated and pre-treatment control groups.

### Excisional Wound Model and Establishment of Infection

The wound healing characteristic of the 4i-PACT was evaluated using a rat model (Dash et al., 2001). **The Animal Care and Use Committee of PUMC approved all experimental protocols involving rats.** Sprague–Dawley rats from Beijing HFK Bioscience CO.,LTD, weighing about 220 g, were housed at one rat one cage and maintained in dark except PACT treatment. The rats were first anesthetized via intraperitoneal injection of chloral hydrate (300 mg/kg). Then their back was shaved with an electric razor, followed by a depilatory agent. Four circular wounds (1.5 cm in diameter) with a depth of the skin layers were prepared along both sides of the spine. Immediately after that, suspension (50 μL) of mixed bacteria (10^9^ CFU/mL MRSA, 5 × 10^8^ CFU/mL *E. coli*, 5 × 10^8^ CFU/mL *P. aeruginosa*) in sterile PBS was inoculated onto the surface of each wound with a pipet tip and then smeared onto the wound surface with an inoculating loop. Next, a bandage was wrapped around each rat to protect the shaved skin and the wounds from other harm. This state was kept for 1 day. Then these rats were used as the wounds model for mixed bacterial infection.

### 4i-PACT *In Vivo*

Thirty rats with wound infected by mixed bacteria were divided into five groups: (A) no treatment control; (B) 4i + 100 J/cm^2^ Light; (C) 4i + 50 J/cm^2^ Light; (D) 4i + 25 J/cm^2^ Light; (E) 4i + 12.5 J/cm^2^ Light. Since light illumination doses alone in this study do not have significant effect on the wound healing, we did not provide light illumination doses alone groups.

Twenty four hours after infection, 50 μL of 4i solution (40 μM in PBS) was injected under eschar of the wound of groups B–E. Then after 30 min for the PS uptaken by the bacteria, rats in groups B–E were illuminated with 650 nm laser (100 mW/cm^2^), the light dose was kept at 100, 50, 25, 12.5 J/cm^2^ for group B–E, respectively. On the next day, the same light dose was given to increase the antibacterial effect of 4i-PACT. The above treatment was repeated three times.

The day, on which rats were infected, was regarded as day 1, the widths and lengths of the wounds were measured using a vernier caliper on days 1, 2, 3, 4, 5, 6, 7, 8, 10, and 12. The viability of each group of rats after infection was recorded, respectively. Meanwhile bacterial CFUs in the wound were calculated in the following ways. Swabs collected from the rat wounds were cultured in PBS. Viable bacteria in the PBS were counted by making 10-fold serial dilutions and culturing the dilutions on LB agar for 18 h at 37°C. Then CFUs were counted manually (Kumar et al., 2011; Sudheesh Kumar et al., 2012).

### Statistical Analysis

All data are presented as mean ± SEM. The significance of differences between sample means was determined by Student’s *t*-test using SPSS 19.0.

## Results and Discussion

### MIC and MBC Determinations

The minimum inhibitory and bactericidal concentration (MIC and MBC) of 4i toward bacteria was studied. Bacterial suspensions (10^6^ CFU/mL) were incubated with the 4i in dark for 30 min at 37°C and then exposed to light illumination (650 nm, 6 J/cm^2^). The concentration of 4i required to make the suspensions (10^6^ CFU/mL) change visibly from turbid to clear was regarded as the minimum inhibitory concentration (MIC), while the concentration at which no more than five colonies were observed on the plates was regarded as the minimum bactericidal concentration (MBC). As shown in **Table 1**, compound 4i had high bacterial photoinactivation ability with MIC values of 3.91 μM against MRSA, 1.95 μM against *E. coli*, and 7.81 μM against *P. aeruginosa*, respectively; MBC values of 7.81 μM against MRSA, 3.91 μM against *E. coli*, and 15.60 μM against *P. aeruginosa*. Meanwhile the dark toxicity of the 4i is relatively weak, with MIC > 62.5 μM, MBC > 125 μM for these three strains. From the values of MBC, it can be concluded that 4i-PACT is very effective toward *E coli*, *P. aeruginosa* is the most resistant strain and MRSA is at the middle of them. So 4i-PACT seemly shows higher bactericidal activity toward Gram-negative strains than that of Gram-positive strains. This trend is different from the PACT of other positive charged PS (Sahu et al., 2009; Kharkwal et al., 2011; Ke et al., 2014), which show higher photoinactivation toward the Gram-positive strains than the Gram-negative counterparts. The latter possess a highly organized outer membrane which imparts a higher permeability barrier for the PSs to penetrate into the bacteria. In this study, 4i-PACT shows higher bactericidal effect toward *E. coli* than MRSA and *P. aeruginosa*. This unexpected result can be interpreted based upon the chemical structure of 4i, π–π interaction of large planar porphyrin rings, hydrogen bonding interaction and charge interaction from four lysine moieties. The ensemble interaction and difference of the outside membrane structure of these strains lead to the unexpected bactericidal effect of 4i-PACT.

**Table 1:** Minimal inhibitory concentration (MIC, μM) and minimal bactericidal concentration (MBC, μM) of 4i against MRSA, *Pseudomonas aeruginosa*, *Escherichia coli.*

Clinical bacterial strain	Light toxicity		Dark toxicity	
	MIC	MBC	MIC	MBC
MRSA	3.91	7.81	62.50	125.00
*P. aeruginosa*	7.81	15.60	250	>500
*E. coli*	1.95	3.91	62.50	125.00

### Dose-Dependent Photoinactivation Effects

**FIGURE 2:**
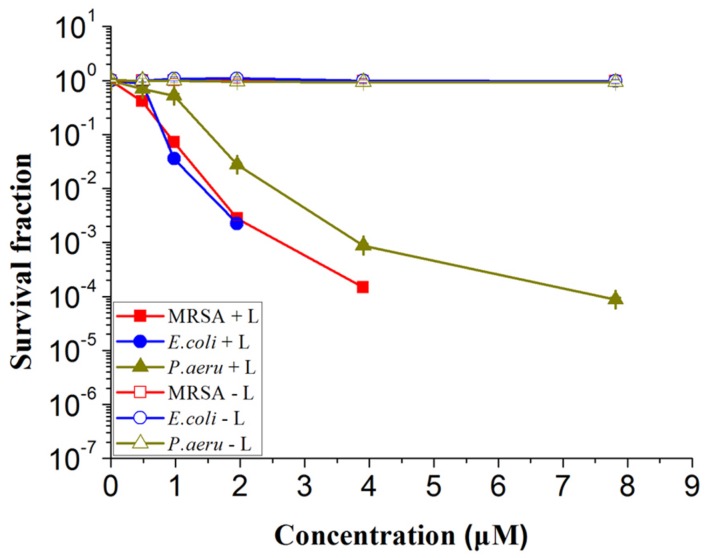
**Survival fraction of three bacterial strains treated by different concentration of 4i with and without illumination.** Light toxicity marked by MRSA + L, *Escherichia coli* + L, *Pseudomonas aeruginosa* + L and dark toxicity marked by MRSA – L, *E. coli* – L, *P. aeruginosa* – L respectively.

The photoinactivation effect of 4i against MRSA, *E. coli* and *P. aeruginosa* strains was dose-dependent (**Figure 2**). A sharp decrease in bacterial survival fraction with increase of the concentration was observed. These three strains were incubated with 3.9 μM 4i in the dark for 30 min at 37°C, then illuminated by the 650 nm laser with power of 6 J/cm^2^. 4.35, 6.96, and 3.56 log_10_ reduction in bacterial survival fraction was observed for the clinical isolated MRSA, *E. coli* and *P. aeruginosa*, respectively. As a control, 4i did not show any bactericidal effect to these strains in dark at less than 10 μM.

### Morphology Changes

**FIGURE 3:**
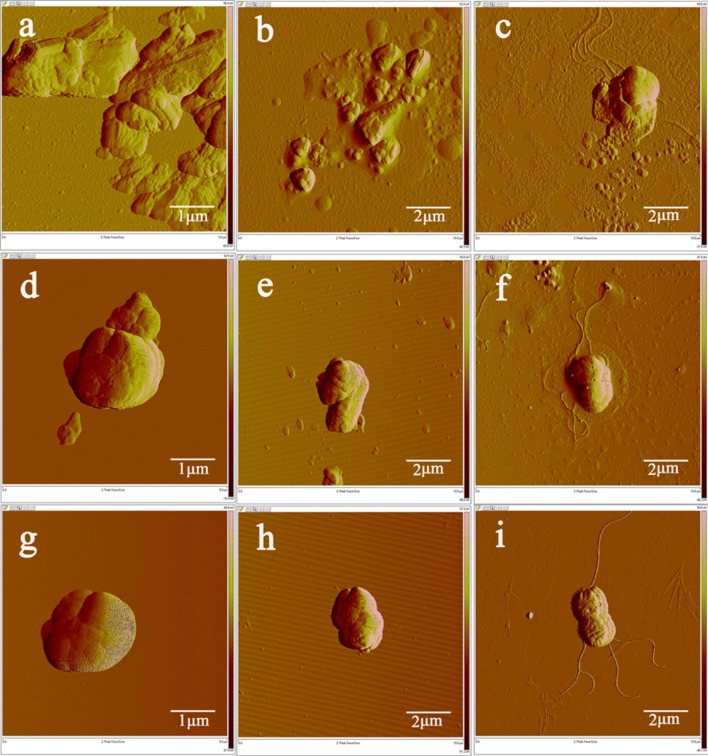
**Fluorescence microscopy image of three bacterial strains treated by 10 μM 4i with and without illumination. (a)** MRSA treated by 4i-PACT, **(d)** 4i with no light, **(g)** control; **(b)** *P. aeruginosa* treated by 4i-PACT, **(e)** 4i with no light, **(h)** control; **(c)** *E. coli* treated by the 4i-PACT, **(f)** 4i with no light, **(i)** control.

Changes of the bacterial structure can be clearly revealed at morphological level by AFM. The representative results are shown in **Figure 3**. The normal ball shape structure of MRSA (**Figure 3g**), the rod-like shape of *P. aeruginosa* (**Figure 3h**) and *E. coli* (**Figure 3i**) was observed without any treatment. The size and shape of the bacteria agree with that reported in previous study (Eaton et al., 2008). For these three strains treated by 4i in the dark (**Figures 3d-f**), it can be seen that the bacterial structure is complete. Particles scattered at the surface of bacteria may be the aggregates of 4i. In the groups treated by 4i-PACT (**Figures 3a–c**), it was found that bacteria is damaged and fragmented. Some irregular aggregation of the dead corpse was detected. Researchers have found that the roughness, size and shape of bacterial strains are slightly altered after PACT treatment with Toluidine Blue O (Sahu et al., 2009; Jin et al., 2010; Lin et al., 2012). The leakage from *Staphylococcus aureus* cell suspension is greatly increased (Sahu et al., 2009), suggesting that cytoplasmic materials are lost through the damaged bacterial membrane. Results in this study showed that bacterial strains are completely broken into pieces. Thus, 4i-PACT treatment appeared to have potent effect on the bacterial envelope, including damaging the bacterial wall and membrane.

### Membrane Integrity

**FIGURE 4:**
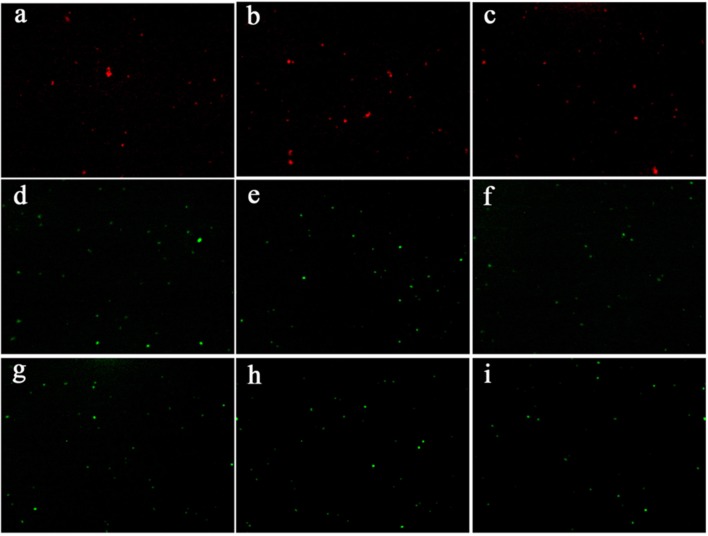
**AFM image of three bacterial strains treated by 10 μM 4i with and without illumination. (a)** MRSA treated by 4i-PACT, **(d)** 4i with no light, **(g)** control; **(b)** *P. aeruginosa* treated by 4i-PACT, **(e)** 4i with no light, **(h)** control; **(c)** *E. coli* treated by 4i-PACT, **(f)** 4i with no light, **(i)** control.

The fluorescence microscopy was used to investigate the permeability of bacterial membrane before and after 4i-PACT. Fluorescent dyes (AO/EB), as the indicator of cell membrane damage, were used to assess the antibacterial activity of 4i-PACT. Living bacteria was observed as green and dead as red, respectively. **Figures 4g–i** show fluorescence microscopy images of the control samples, where no 4i was added, the bacteria is dispersed and alive with green color. **Figures 4a–c** are the fluorescence microscopy images of MRSA, *P. aeruginosa* and *E. coli* treated by 4i-PACT (10 μM, 6J/cm^2^, 650 nm), respectively, where most of the bacteria is dead with red color. On the other hand, fluorescence microscopy images of MRSA (**Figure 4d**), *P. aeruginosa* (**Figure 4e**), and *E. coli* (**Figure 4f**) treated by10 μM 4i without illumination showed little bactericidal effect. The above results mean that 4i-PACT can damage the membrane of bacteria and change the permeability of the bacterial membrane. This again explains why the antimicrobial effect of 4i-PACT is so strong.

### Photodynamic Effect on Genomic DNA

**FIGURE 5:**
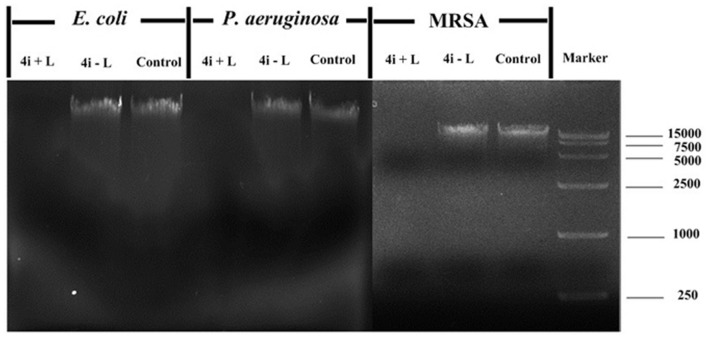
**Agarose gel electrophoresis of three bacterial strains after treated by 10 μM 4i with and without illumination.** Bacteria treated with 4i-PACT (4i + L), treated with 4i in the dark (4i–L) and no treatment (control).

The influence of 4i-PACT on bacteria was further studied from a genomic point of view such as DNA leakage or damage. The electrophoresis graph (**Figure 5**) shows that the DNA bands, extracted from the processed bacterial cells, disappear after 4i-PACT (10 μM, 6 J/cm^2^, 650 nm), while a clear DNA band is detected in the control groups with no 4i-PACT or treated by 4i alone. This result is consistent with the AFM morphological study as well as the fluorescent staining, which reveal that the 4i-PACT breaks the bacterial membrane and wall and causes the leakage of genomic DNA. The absence of DNA bands can be attributed to either the leakage of bacteria membrane or the break of the DNA. So we conducted another independent experiment. The naked DNA extracted from each strains was treated by 4i-PACT. The agarose gel electrophoresis graph still shows no DNA bands. This result indicated that 4i-PACT can break DNA into pieces. This phenomenon is consistent with the previous results (Menezes et al., 1990; Choi et al., 2010; Lin et al., 2012). Generally, the reason for multidrug resistance in cellular level is due to the drug only blocked or weakened the partial binding site or signal pathway, while the whole cellular bodies were kept, so it is easy to develop multidrug resistance. Double effects of 4i-PACT, destroying bacterial membrane and damaging the DNA, can account for the vanish of DNA band in electrophoresis graph. This is the reason why bacterial strains have no resistant to 4i-PACT.

### Bacterial Content Exudation

Release of intracellular components is a good indicator of membrane integrity. Small ions such as potassium and phosphate tend to exude first, followed by large molecules such as DNA, RNA, and other materials. Since these nucleotides have strong UV absorption at 260 nm, measuring the absorbance at 260 nm can reveal the information of membrane integrity (Denyer and Hugo, 1991; Chen and Cooper, 2002). As shown in **Table 2**, after 10 μM 4i-PACT, the bacterial content exudation from MRSA, *P. aeruginosa*, and *E. Coli* are increased by 247.87, 188.98, 336.67%, respectively, compared with pre-treatment samples, while changes less than 10% was observed in the control or 4i group. This result is consistent with that of AFM and fluorescent images. These data are larger than that reported by Sahu et al. (2009), which were only 20–60% increase compared with the control groups.

**Table 2:** Bacterial content exudation in the control (PBS), 4i alone, and 4i-PACT treatment groups (*n* = 4, mean ± SD) measured at 260 nm.

Clinical bacterial strain	Increase in OD_260_ relative to pre-treatment (%)		
	Control	4i alone	4i-PACT
MRSA	2.86 ± 0.34	10.98 ± 0.41	247.87 ± 17.87
*P. aeruginosa*	2.04 ± 0.29	9.54 ± 0.28	188.98 ± 19.32
*E. coli*	2.98 ± 0.33	13.31 ± 0.38	336.67 ± 28.08

### *In Vivo* Antibacterial Activity and Wound-Healing Effect

**FIGURE 6:**
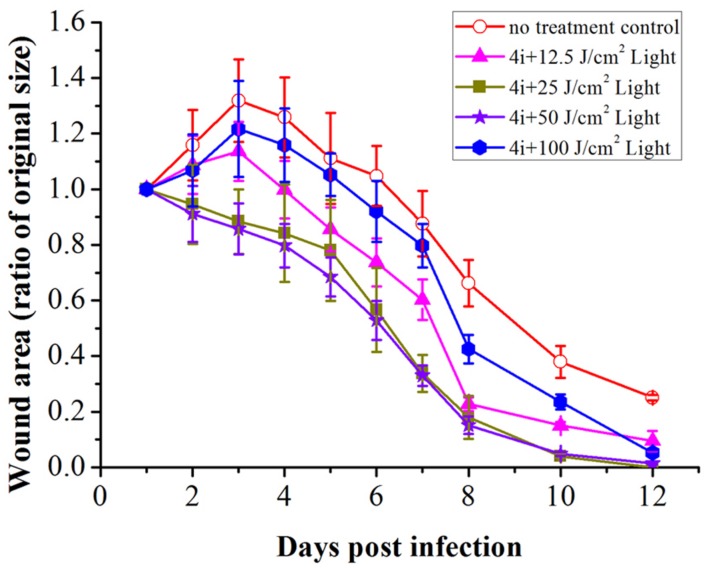
**Wound healing ratio over time in bacterial infected rats with and without 4i-PACT.** Each point represents the mean ratio of the area of the original wound. Data is expressed as mean ± SEM. *N* = 6 in each group.

*In vivo* antibiotics experiment, generally 5 or 10 times MIC was chosen as the working concentration. 40 μM of 4i, five times of MIC (Singh et al., 2006; Dai et al., 2009) was chosen as the working dose. The antibacterial effect of 4i-PACT with different light dose was studied. The wound healing rate is highly light dose dependent (**Figure 6**). Control group A, with no treatment, showed less wound healing at each observation time point. A rapid wound healing was observed in the treatment of groups C (50 J/cm^2^) and D (25 J/cm^2^) during the day 4 to 8 after infection (*p* < 0.05). After day 8, wound healing became slow till completely healed on day 12. However, in the groups B (100 J/cm^2^) and E (12.5 J/cm^2^), rapid wound closure happened from day 7 to 10. On the day 8, mean wound area ratios of B–E were 0.4252 ± 0.0512, 0.1515 ± 0.0312, 0.1823 ± 0.0777, and 0.2273 ± 0.0239, respectively, but in the control group wound area ratio was 0.6619 ± 0.0834, indicating B–E groups had better wound healing ratio compared with the control group. Almost total wound closure was observed by the day 12 post infection in all treated groups.

**FIGURE 7:**
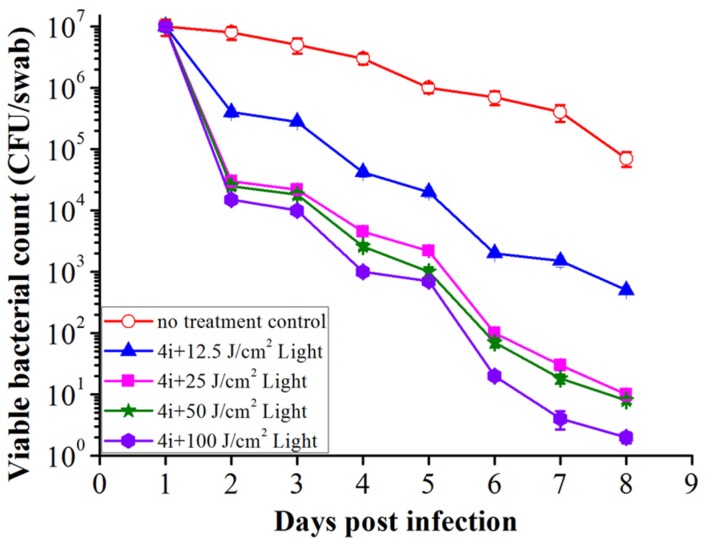
**The mean bacterial counts of the swabs collected from the rat wounds during the 8 days post infection.** Data is expressed as mean ± SEM. *N* = 6 in each group.

The viable bacteria in wound tissue were counted as an index of the bactericidal effect of 4i-PACT. On days 1, 2, 3, 4, 5, 6, 7, 8 post infection, PACT-treated groups exhibited obvious reduction in bacterial viability compared with the no treatment control group (**Figure 7**). At the same time point, the decrease of viable bacterial count is in the order of light illumination dose, B > C > D > E. This suggests that 4i-PACT accelerates wound healing via bactericidal effect against MRSA, *P. aeruginosa*, and *E. coli*. However, the wound healing ratio is in the order of C > D > E > B, instead of paralleling of the bactericidal effect. This indicates that 4i-PACT effect on normal tissue should be considered. A rationalized interpretation can be given based upon the multiple function of 4i-PACT *in vivo*: 4i-PACT not only photoinactivates bacteria, but also causes the normal tissue damaged and repaired. The observed effect is the balance of the ensemble results. For either a weaker 4i-PACT with a lower illumination dose (12.5 J/cm^2^ in group E), or a stronger 4i-PACT with a higher illumination dose (100 J/cm^2^ in group B), the total results are not good. The best result is from treatment with medium light dose like group C (50 J/cm^2^). So for the practical application of PACT further study is requires.

**FIGURE 8:**
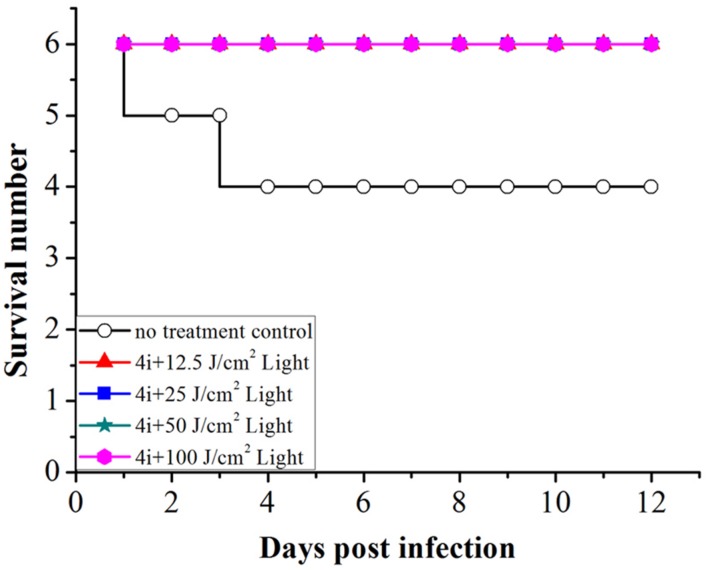
**The viability of rats against days post infection for treated versus not treated**.

In the no treatment control group, two rats died on day 2 and day 4, rats in other groups all survived the experiment. Therefore, the viability of rats post treatment indicates that 4i-PACT is more efficient in the treatment of mixed bacterial infection (**Figure 8**).

## Conclusion

This paper studied the photoinactivation mechanism of 4i-PACT *in vitro* and its wound healing effect on wound rat model infected by mixed bacteria. 4i-PACT (3.91 μM, 6J/cm^2^, 650 nm) had a broad potent antibacterial spectrum: 4.35, 6.96, and 3.56 log_10_ reduction in bacterial survival for MRSA, *E. coli*, and *P. aeruginosa*, respectively. AFM revealed that 4i-PACT can destroy bacterial wall and membrane. This is further confirmed by Dual fluorescent staining with AO/EB. Agarose gel electrophoresis indicated that 4i-PACT can damage the genomic DNA of bacteria. Absorbance at 260 nm reveals that 4i-PACT could cause bacterial content leakage. 4i-PACT breaks not only bacterial wall but also the genomic DNA. That is why the bacterial strains have no resistance to 4i-PACT. Wound healing effect on mixed bacterial infected rat model revealed that 4i-PACT *in vivo* is highly potent and light-dose dependent. The ensemble effect is the balanced effect of 4i-PACT both to normal tissue and bacterial strains. The best light dose is 50 J/cm^2^. Overall, 4i-PACT is a highly efficient treatment modality for bacterial infection *in vitro* or *in vivo*.

## Author Contributions

TL designed experiments; SM, ZX, LP, and YG carried out experiments; YG and ZX analyzed experimental results, CW analyzed sequencing data. TL, ZX, and YG wrote the manuscript. TL designed experiments; SM, ZX, LP, and YG carried out experiments; YG and ZX analyzed experimental results, CW analyzed sequencing data. TL, ZX, and YG wrote the manuscript.

## Conflict of Interest Statement

The authors declare that the research was conducted in the absence of any commercial or financial relationships that could be construed as a potential conflict of interest.
